# Hand grip strength as a surrogate marker for postoperative changes in spinopelvic alignment in patients with lumbar spinal stenosis

**DOI:** 10.1038/s41598-020-70357-8

**Published:** 2020-08-10

**Authors:** Ji-Won Kwon, Byung Ho Lee, Sahyun Sung, Soo-Bin Lee, Moon-Soo Park, Jun-Hee Cho, Jae-Ho Yang, Hwan-Mo Lee, Seong-Hwan Moon

**Affiliations:** 1 Department of Orthopedic Surgery, Yonsei University College of Medicine, 50 Yonsei-ro, Seodaemun-gu, Seoul, 120-752 Korea; 2 Department of Orthopedic Surgery, Hallym University College of Medicine, Gyeonggi-do, Korea; 3 Department of Orthopedic Surgery, National Health Insurance Service Ilsan Hospital, Goyang, Korea

**Keywords:** Quality of life, Risk factors

## Abstract

There are a few studies on the postoperative changes in sagittal alignment and corresponding factors, including hand grip strength (HGS) and muscle performance tests for lumbar spinal stenosis (LSS). Thus, we aimed to determine whether HGS can be a surrogate marker for global sagittal alignment changes after decompression with fusion surgery for LSS. This retrospective observational study included 91 patients who underwent spine fusion surgery for LSS. Radiological spinopelvic parameters, including sagittal vertical axis (SVA), lumbar lordosis (LL), pelvic tilt (PT), pelvic incidence (PI), global tilt (GT), and T1 pelvic angle (T1PA), were analyzed preoperatively and 1 year after posterior decompression and fusion surgery. To assess muscle performance, the 6-m walk (SMT), timed up and go (TUGT), and sit-to-stand (STS) tests were conducted. The relationship between HGS and postoperative SVA was examined through multiple linear regression analysis. Additionally, the relationship between HGS and preoperative/postoperative radiologic spinopelvic parameters and muscle performance test results was analyzed through Pearson's correlation. HGS was significantly correlated with age, preoperative and postoperative SVA, and the muscle performance tests. Furthermore, HGS was a factor that can significantly influence postoperative SVA changes in multiple linear regression analyses. Therefore, HGS may be a good predictor of postoperative SVA change.

## Introduction

Sarcopenia is an aging-related phenomenon characterized by the generalized loss of skeletal muscle mass. The current consensus regarding the definition of sarcopenia is low skeletal muscle mass and function according to the European Working Group on Sarcopenia in Older People (EWGSOP)^1,2^. For the diagnosis of sarcopenia, the EWGSOP recommends the presence of both low muscle mass and low muscle function (strength or performance). The muscle strength that makes up the skeletal muscle function can be measured using a hand grip strength (HGS) dynamometer and is a measure of voluntary muscle function^3^. One of the other factors, physical performance can be assessed by several gait speed tests, including the 6-m walk (SMT), timed up and go (TUGT), and sit-to-stand (STS) tests. Similarly, lumbar spinal stenosis (LSS) could result from aging and degenerative processes, such as sarcopenia. In fact, LSS is significantly associated with sarcopenia, and the conditions tend to coexist^4^. Driven by this trend, there have been several attempts to estimate which factors could affect LSS treatment outcomes, such as spinopelvic parameters and other tools of clinical outcomes^5,6^. This study was planned with the aim of identifying the mutual relationship between two disease entities, sarcopenia and spinal stenosis, which are essential characteristic aspects of the aging process. Therefore, this study aimed to examine the relationship between radiologic spinopelvic parameters (after the surgical treatment of patients with LSS) and skeletal muscle function, that is, the results of HGS and muscle performance tests.

## Methods

### Subjects

This study was approved by our Institutional Review Board and Ethics Committee (Yonsei University Institutional Review Board and Ethics Committee: 2018-2116-001), which issued a waiver regarding the need for informed consent. All experiments were performed in accordance with relevant guidelines and regulations. Between April 2014 and July 2015, 91 patients (43 men, 48 women) with LSS underwent lumbar spine surgery, including posterior decompression and fusion. No patient was diagnosed with hand and wrist-related diseases or cervical spine problems, and all patients were identified for instability or a need for resection > 50% of the facet joints owing to foraminal stenosis and degenerative spondylolisthesis of > grade 2^7,8^. All patients were treated with posterior decompression and posterolateral fusion using local autologous and allo-chip bone grafts and instrumentation. Similarly, other basic demographic data, including age, sex, body mass index (BMI), the existence of osteoporosis, and history of falling, were gathered. The history of falling screening prompted patients or caregivers to write a fall diary each time they visited an outpatient clinic by recording the number of falls in the previous 3 months.

### Hand grip strength

Hand grip strength was measured using a JAMAR plus + hand-grip dynamometer (Global Medical Devices, Maharashtra, India). The patients were instructed to squeeze the handle as hard as possible for 3 s, and the maximum contractile force was recorded in kilograms. The tests were performed three times on both hands, and the respective highest values were used in the analysis^9^.

### The assessment of physical performance

Three functional mobility tests, which were validated by numerous previous studies for the evaluation of muscle physical performance were conducted as follows: Six-Meter-Walk Test (SMT), Sit-to-Stand test (STS), and Timed Up and Go test (TUGT). SMT measures the time to complete six meters along a single location at normal walking speed. Patient performance was timed from the moment their foot crossed the start line to the moment their foot crossed the stop line. If patients did not follow the instructions, the observer stopped the patients to try again. In STS, the patients sat on a standard chair (43-cm) with arms folded and subsequently stood up. The motion is defined as one cycle. The total time taken from 5 repeated cycles is the value of STS. TUGT measures the time while patients get up from an armchair (approximate chair height of 46 cm), walk along a line of three meters on the floor, turn around, and walk back to sit down^10^.

### Radiological measurement and classification into several groups

Standard full-length 36-in. lateral radiographs of the spine were used to assess whole-spine sagittal alignment. The whole-spine lateral radiographs were evaluated before surgery and one year after surgery. The sagittal vertical axis (SVA), thoracic kyphosis (TK), thoracolumbar kyphosis (TLK), lumbar lordosis (LL), pelvic tilt (PT), pelvic incidence (PI), sacral slope (SS), global tilt (GT), pelvic incidence minus lumbar lordosis (PI-LL), and T1 pelvic angle (T1PA) were measured as previously described. SVA was defined as the distance between the C7 plumb line and the posterosuperior corner of the sacrum. Using the Cobb method, LL was measured between the superior endplate of L1 and superior endplate of S1, and TK was measured between the superior endplate of T5 and the inferior endplate of T12. TLK was measured as the angle between the upper endplate of T10 and the lower endplate of L2^11,12^. SS is the angle of the sacral plateau to the horizontal, whereas PT is the angle between a vertical line and the line connecting the center of the femoral head to the center of the sacral plateau of S1. PI is the algebraic sum of the two angles: PT and SS. T1PA was defined as the angle subtended by a line from the femoral head to the center of the T1 vertebral body and a line from the femoral head to the center of the superior sacral endplate. GT was defined as the angle subtended by a line from the center of the superior sacral endplate to the center of the C7 vertebral body and a line from the femoral head to the center of the superior sacral end plate^13,14^.

### Statistical analysis

Categorical variables were analyzed using the chi-square test, whereas continuous variables were analyzed using Student’s t-test to evaluate the differences between the pre- and postoperative measurements. Pearson’s correlations between HGS, radiologic parameters, and results of physical performance tests were evaluated. To test the hypothesis that HGS might be independently associated with postoperative SVA, multiple linear regression analyses were performed using an enter method with HGS as the independent variable and postoperative SVA as the dependent variable. Potential confounding factors, such as age, sex, BMI, presence of osteoporosis, history of falling, SMT, TUGT, and STS, were similarly considered independent variables. All statistical analyses were performed using SPSS Statistics 23 (IBM Corp., Chicago, IL, USA). *P* values < 0.05 were considered statistically significant.

## Results

### Patient demographics

Ninety-one patients (43 men and 48 women) were enrolled in this study. The mean patient age was 68.0 years (range, 33–86 years). Other demographic descriptions, including sex, BMI, and the existence of osteoporosis, are shown in Table 1.

**Table 1:** Characteristic of the enrolled patients.

Number of patients	91
Mean age (yrs)	68
Sex (Male/Female)	43/48
BMI	24.67
History of falling (number of falling in the last three months)	1.4
Osteoporosis (Y/N)	18/73
Decompression and fusion level	1.8
Hand grip strength (kg)	17.3

Values are expressed in mean and standard deviation.

*BMI* body mass index.

### Pre- and postoperative changes in radiographic/clinical parameters

The pre- and postoperative radiographic/clinical parameters are shown in Table 2. No significant differences were observed in SVA, LL, PT, SS, PI-LL, TK, TLK, GT, and T1PA or history of falling. However, significant differences were observed in TUGT, SMT, and STS, with more improvement in performance at 1-year postoperative follow-up than before surgery.

**Table 2:** Preoperative and postoperative radiologic and clinical parameters.

Parameters	Preoperative	Postoperative	*P* value
SVA	51.1 ± 43.0	44.2 ± 42.8	0.133
LL	35.9 ± 15.3	37.6 ± 15.1	0.149
PT	22.1 ± 8.2	22.8 ± 9.6	0.329
SS	33.0 ± 9.0	31.7 ± 9.5	0.177
PI	55.1 ± 11.5	54.6 ± 10.0	0.575
PI-LL	19.2 ± 15.6	17.0 ± 14.9	0.111
TK	17.5 ± 9.3	18.8 ± 10.8	0.057
TLK	9.6 ± 7.3	8.9 ± 6.7	0.144
GT	27.4 ± 10.8	27.3 ± 13.4	0.964
T1PA	22.9 ± 9.4	22.5 ± 11.1	0.682
History of falling	1.0 ± 2.4	1.1 ± 6.2	0.902
TUGT	16.3 ± 7.3	12.0 ± 4.8	< 0.001*
STS	20.5 ± 6.7	16.4 ± 6.3	< 0.001*
SMT	12.3 ± 6.1	9.4 ± 4.8	< 0.001*
SVA	51.1 ± 43.0	44.2 ± 42.8	0.133

*SVA* sagittal vertical axis, *LL* lumbar lordosis, *PT* pelvic tilt, *SS* sacral slope, *PI* pelvic incidence, *PI-LL* pelvic incidence minus lumbar lordosis, *TK* thoracic kyphosis, *TLK* thoracolumbar kyphosis, *GT* global tilt, *T1PA* T1 pelvic angle, *TUGT* timed up and go test, *STS* sit-to-stand test, *SMT* 6-m walk test.

* means *P* value is within 0.05

### Correlation between HGS and demographic/radiologic parameters and clinical outcomes

HGS was correlated with preoperative SVA, PT, PI, GT, T1PA, PI-LL, and TK. Similarly, HGS was correlated with postoperative SVA, LL, PT, PI, GT, T1PA, PI-LL, and TK. In terms of demographic and radiologic parameters, HGS was correlated with age, postoperative SMT, and TUGT (*P* < 0.05 for all) (Tables 3 and 4).

**Table 3:** Correlation between HGS and demographic/clinical parameters.

		History of falling (n)	SMT(s)	TUGT(s)	STS (s)	Age (yr)
**Preoperative**						
HGS	Pearson’s coefficient (R)	− 0.135	− 0.196	− 0.156	− 0.038	− 0.381*
	P value	0.202	0.063	0.139	0.723	< 0.001
**Postoperative**						
HGS	Pearson’s coefficient (R)	− 0.019	− 0.377*	− 0.368*	− 0.164	
	P value	0.859	< 0.001	< 0.001	0.120	

*HGS* hand grip strength, *SMT* 6-m walk test, *TUGT* timed up and go test, *STS* sit-to-stand test.

* means *P* value is within 0.05

**Table 4:** Correlation between HGS and preoperative/postoperative radiologic parameters.

		SVA	LL	PT	SS	PI	GT	T1PA	PI-LL	TK	**TLK**
**Preoperative**											
HGS	Pearson’s coefficient (R)	− 0.375*	0.090	− 0.277*	− 0.031	− 0.223*	− 0.360*	− 0.345*	− 0.252*	0.279*	-0.170
	P value	< 0.001	0.395	0.008	0.770	0.033	< 0.001	0.001	0.016	0.007	0.107
**Postoperative**											
HGS	Pearson’s coefficient (R)	− 0.394*	0.226*	− 0.330*	0.079	− 0.242*	− 0.431*	− 0.407*	− 0.391*	0.222*	-0.190
	P value	< 0.001	0.031	0.001	0.458	0.021	< 0.001	< 0.001	< 0.001	0.035	0.071

*HGS* hand grip strength, *SVA* sagittal vertical axis, *LL* lumbar lordosis, *PT* pelvic tilt, *SS* sacral slope, *PI* pelvic incidence, *GT* global tilt, *T1PA* T1 pelvic angle, *PI-LL* pelvic incidence minus lumbar lordosis, *TK* thoracic kyphosis, *TLK* thoracolumbar kyphosis.

* means *P* value is within 0.05

### Determination of covariates independently associated with postoperative SVA

Multiple linear regression analyses were performed to determine which covariates, including HGS, were independently associated with postoperative SVA. Only HGS tended to be significantly inversely related to postoperative SVA (Table 5) (p < 0.05).

**Table 5:** Multiple linear regression analysis to identify covariates associated with SVA.

Independent variable	Dependent variable: postoperative SVA		
	β	SE	*P*
Age	0.065	0.605	0.575
Sex	0.014	9.855	0.901
BMI	0.111	1.386	0.297
Presence of osteoporosis	0.038	11.228	0.722
History of falling	0.085	1.892	0.418
Preoperative SMT	0.269	1.245	0.133
Preoperative TUGT	-0.169	1.056	0.349
Preoperative STS	-0.014	0.737	0.902
HGS	-0.360	0.690	0.006*

The enter method was applied to this model with HGS as an independent variable and age, BMI, presence of osteoporosis, history of falling, and preoperative SMT/TUGT/STS as dependent variables simultaneously.

*SVA* sagittal vertical axis, *β* regression coefficient, *SE* standard error, *BMI* body mass index, *SMT* 6-min walk test, *TUGT* timed up and go test, *STS* sit to stand test, *HGS* hand grip strength.

## Discussion

The changes in the sagittal alignment of patients with LSS were investigated after posterior decompression and fusion surgery; additionally, the correlations of HGS with demographic, radiological, and clinical parameters were analyzed, respectively. As sarcopenia is a result of the aging process, HGS was observed to be inversely correlated with age^1,2^. In the initial study design, we focused on the mechanism underlying the improvement of the patient’s sagittal alignment as a neurological problem, including the assessment of changes in back pain and claudication after surgery. We hoped that the HGS would be an index of preoperative evaluation because it may affect the muscle performance and radiologic parameters, including LL, TLK, PT, GT, PI-LL, T1PA, after surgery.

For the postoperative evaluation, the follow-up period of postoperative clinical outcomes or radiologic parameters could be set to more than two years. However, the period for postoperative follow-up was set as one year because HGS is expected to have a constant value within a year^15,16^. As a diagnostic tool for sarcopenia, we aimed to ascertain the mechanism underlying the effect of HGS on the sagittal alignment and muscle performance in patients with LSS briefly after surgery. Therefore, we did not set the follow-up duration longer to maintain a relatively constant effect for HGS on postoperative clinical outcomes and sagittal alignment.

Moreover, HGS and the postoperative radiologic parameters were observed to be significantly related to and could affect the postoperative global sagittal alignment^17^. Similarly, HGS was associated with postoperative muscle performance and preoperative/postoperative clinical outcomes. Multiple studies, to date, have shown a correlation between clinical outcomes and preoperative HGS after hip fracture, esophageal cancer, and degenerative spinal stenosis surgery, wherein cases with a high HGS demonstrated better surgical outcomes^18–20^. These results are similar to those of this study evaluating the correlation between HGS and surgical outcomes in LSS. This study presents similar conclusions regarding clinical outcomes, including muscle performance tests. In this regard, HGS was observed to be a significant influencing factor, and this result suggests that patient-specific HGS is a major influencing factor of postoperative muscle performance. To determine HGS as a predictor of postoperative sagittal alignment, multiple linear analyses were performed. For all covariates except HGS, no statistically significant associations were observed with postoperative SVA. Thus, HGS may be a factor indicating global sagittal alignment that contributes to muscle performance, such as the paraspinal muscles, balancing the global sagittal alignment^3,4,15,21^. A decrease in muscle strength or performance status implies that the sagittal alignment is likely unbalanced in older patients due to the aging process, consistent with the definition of sarcopenia. Supposedly, this study provides useful implications that HGS could serve as a useful predictor of patient outcomes. This study has several limitations. First, it has a retrospective design and was conducted without correction or control of the sex ratio. Second, this study has a relatively small sample size and a short follow-up period of 1 year. To better establish the predictive value of HGS for LSS, a prospective study with large sample size and long-term follow-up is necessary to confirm these findings.

## Conclusion

Therefore, these findings suggest that HGS can influence surgical outcomes and postoperative sagittal radiologic parameters in patients with LSS and that preoperative HGS may be a good predictor of postoperative SVA status.
